# Parameter identification of sound absorption model of porous materials based on modified particle swarm optimization algorithm

**DOI:** 10.1371/journal.pone.0250950

**Published:** 2021-05-04

**Authors:** Xiaomei Xu, Ping Lin

**Affiliations:** College of Automobile and Traffic Engineering, Nanjing Forestry University, Nanjing, China; Torrens University Australia, AUSTRALIA

## Abstract

Porous materials have been widely used in the field of noise control. The non-acoustical parameters involved in the sound absorption model have an important effect on the sound absorption performance of porous materials. How to identify these non-acoustical parameters efficiently and accurately is an active research area and many researchers have devoted contributions on it. In this study, a modified particle swarm optimization algorithm is adopted to identify the non-acoustical parameters of the jute fiber felt. Firstly, the sound absorption model used to predict the sound absorption coefficient of the porous materials is introduced. Secondly, the model of non-acoustical parameter identification of porous materials is established. Then the modified particle swarm optimization algorithm is introduced and the feasibility of the algorithm applied to the parameter identification of porous materials is investigated. Finally, based on the sound absorption coefficient measured by the impedance tube the modified particle swarm optimization algorithm is adopted to identify the non-acoustical parameters involved in the sound absorption model of the jute fiber felt, and the identification performance and the computational performance of the algorithm are discussed. Research results show that compared with other identification methods the modified particle swarm optimization algorithm has higher identification accuracy and is more suitable for the identification of non-acoustical parameters of the porous materials. The sound absorption coefficient curve predicted by the modified particle swarm optimization algorithm has good consistency with the experimental curve. In the aspect of computer running time, compared with the standard particle swarm optimization algorithm, the modified particle swarm optimization algorithm takes shorter running time. When the population size is larger, modified particle swarm optimization algorithm has more advantages in the running speed. In addition, this study demonstrates that the jute fiber felt is a good acoustical green fibrous material which has excellent sound absorbing performance in a wide frequency range and the peak value of its sound absorption coefficient can reach 0.8.

## 1 Introduction

Nowadays porous materials have been widely used in the field of noise control. Many researchers have devoted contributions on the sound absorption performance of these materials. The sound absorption performance of materials is commonly characterized by the sound absorption coefficient (SAC). The SAC of materials can be experimentally evaluated using the impedance tube [1] or be predicted using acoustic transfer analysis method along with experimental measurements [2]. Allard and Attala demonstrated the prediction of the SAC of porous materials by using the transfer matrix method [3].

There are two main models for porous materials to predict their SAC in previous studies [4]: the empirical model represented by Delany-Bazley (DB) model [5] and the phenomenological model represented by Johnson-Champoux-Allard (JCA) model [6]. The empirical model only needs to measure the air flow resistivity and then establish respectively the power-law relations between the characteristic impedance and the air flow resistivity, and the relations between the propagation constant and the air flow resistivity by fitting a large number of measurements. It is obvious that the empirical models are easy to implement. However, the empirical model does not consider the microstructure of the pores, and moreover, each empirical model is usually best suitable for certain type of materials and certain frequency ranges. The phenomenological model takes the influence of micro-factors on the acoustical properties of the materials into account. They consider the frame of a porous material as rigid and involve five non-acoustical parameters for the surface impedance calculation, namely porosity, tortuosity, air flow resistivity, viscous and thermal characteristic lengths [7]. The phenomenological model establishes a relationship between the microstructure and the acoustic performance through characterizing porous materials with equivalent fluid, which makes them have higher prediction accuracy.

As a representative of the phenomenon model, the JCA model is now the most widely used model in predicting the SAC of porous materials [8]. The prediction accuracy of the SAC considerably depends on the measurement precision or identification precision of the material non-acoustical parameters [9]. Measurement of the non-acoustical parameters is not a simple task, as it involves dedicated measurement facilities that are not very common to all acoustic characterization laboratories [10]. In that case the inverse acoustic characterization method is adopted by many researchers to identify the non-acoustical parameters. The main focus of the method is on the reduction of error between the experimental data and the theoretically predicted data.

Many optimization techniques have been adopted to perform the inverse acoustic characterization method in recent years. Some researchers used traditional optimization techniques like least squares technique [11]. Atalla and Panneton solved the inverse characterization problem of three parameters in the JCA model based on differential evolution algorithm [12]. Pelegrinis et al. used the Nelder-Mead simplex optimization method to solve the error minimization problem [13]. Cobo et al. combined four models and simulated the annealing algorithm to retrieve non-acoustical parameters of the granular acoustic absorbing materials [14]. Bonfiglio and Pompoli compared the effect of different methods applied to determine the physical parameters of porous materials [15]. The research results of literature [15] show that the analytical method and the iterative method are difficult to deal with the non-linear constrains and the optimization solution time of the iterative method is relatively long, the quality of the optimal results for the Nelder-Mead simplex optimization depends considerably on the setting of initial parameters, and the local searching ability of the genetic algorithm is relatively poor and it involves complicated encoding and decoding process.

The particle swarm optimization (PSO) algorithm is also one of the methods used for the inverse characterization problems [16]. The PSO algorithm presents many advantages over other algorithms as it is robust and suitable for the nonlinear design space and it can easily handle continuous, discrete and integer variable types. As a population-based optimization algorithm the PSO algorithm requires lower computational effort [17]. Bansod and Mohanty performed the inverse estimation for the non-acoustical parameters of the jute material with the PSO algorithm and obtained good results of the inverse estimation [17]. However, it is worth noting that for some large-scale nonlinear optimization problems, PSO algorithm is easy to encounter a local optimum solution. In order to avoid this issue, some improvements and modifications have been proposed. The modification approach of PSO algorithm could be grouped as initial solution settings, solution space deduction, evolution process improvement, and heuristic rule, etc. [18].

In this study, a modified particle swarm optimization (MPSO) algorithm is adopted to identify the non-acoustical parameters of the jute fiber felt. The main contributions of this paper are twofold: (1) The identification performance and computation performance of the MPSO algorithm applied to identifying the non-acoustical parameters of the natural porous materials are explored; (2) The sound absorption performance of the natural jute fiber felt in a wide frequency range is revealed.

The remainder of this article is organized as follows. A sound absorption model of porous materials is introduced in Section 2. In Section 3, a non-acoustical parameter identification model is established. In Section 4, a modified particle swarm optimization algorithm is illustrated. And in Section 5, application of the modified particle swarm optimization algorithm in the non-acoustical parameters identification of the jute fiber felt is investigated and discussed. In the last section, concluding remarks are provided and directions for future work are highlighted.

## 2 Sound absorption model

As the semi-empirical model, JCA model is the most widely used sound absorption model. It contains five physical parameters, namely that, porosity *ϕ*, air flow resistivity *σ*, tortuosity *α*_∞_, viscous characteristic length Λ and thermal characteristic length Λ’. Porosity is the percentage of pore volume occupied by saturated medium (generally air) compared to the total volume of the material in the natural state. Air flow resistivity has important influence on the sound absorption performance of porous materials. It is usually defined as the resistance of air flowing through the porous material with certain thickness. Tortuosity of porous materials is the deviation between the actual path and the straight path of the sound waves in materials, which represents the complexity of the material pores. Both the porosity and the tortuosity are dimensionless quantities. The viscous characteristic length represents the magnitude of the viscous force and the thermal characteristic length describes the degree of thermal exchange between the saturated medium in the pore and the solid frame at high frequencies.

According to the JCA model, the effective density *ρ*_*e*_(*ω*) and bulk modulus *K*_*e*_(*ω*) of the porous materials can be calculated using the following expressions.

$$\rho_{e}(\omega )=\alpha_{\infty }\rho_{0}[1-j\frac{\sigma \phi }{\alpha_{\infty }\rho_{0}\omega }\sqrt{1+j\frac{4\alpha_{\infty }^{2}\rho_{0}\omega \eta }{\sigma^{2}\phi^{2}\Lambda^{2}}}]$$ (1)

$$K_{e}(\omega )=\gamma P_{0}{\left[\gamma -\frac{\gamma -1}{1-j\frac{8\eta }{\omega \rho_{0}N_{pr}\Lambda^{\prime 2}}{\left(1+j\frac{\rho_{0}\omega \Lambda^{\prime 2}N_{pr}}{16\eta }\right)}^{1/2}}\right]}^{-1}$$ (2)

where *ω* is the angular frequency of the incident wave, *j* is the imaginary unit, *ρ*_0_ is the air density, *N*_*pr*_ is the Prandtl number of air, *η* is the dynamic viscosity of air, *γ* is the specific heat ratio related to the air state, and *P*_0_ is the ambient atmospheric pressure. It needs to be noted that Λ and *Λ’* are associated with some other physical parameters of the materials and they can be written as

$$\Lambda =\frac{1}{c}{\left(\frac{8\alpha_{\infty }\eta }{\sigma \phi }\right)}^{1/2}\;\Lambda \prime =\frac{1}{c^{\prime }}{\left(\frac{8\alpha_{\infty }\eta }{\sigma \phi }\right)}^{1/2}$$ (3)

where *c* and *c*´ are shape factor and scale factor of the pore cross section, respectively.

The characteristic impedance *Z*_*c*_ (*ω*) and complex propagation constant *k*_*e*_ (*ω*) of porous materials can be deduced by Eqs (1) and (2), and they can be expressed as Eqs (4) and (5).

$$Z_{c}(\omega )=\sqrt{K_{e}(\omega )\rho_{e}(\omega )}$$ (4)

$$k_{e}(\omega )=\omega \sqrt{\frac{\rho_{e}(\omega )}{K_{e}(\omega )}}$$ (5)

Considering that the porous material with thickness *d* is backed by the rigid boundary, the sound absorption coefficient (SAC) *α* of the porous material can be denoted by the following equations.

$$Z_{s}(\omega )=-j\frac{Z_{c}(\omega )}{\phi }\text{cot}(k_{e}(\omega )d)$$ (6)

$$R=\frac{Z_{s}(\omega )-Z_{0}}{Z_{s}(\omega )+Z_{0}}$$ (7)

$$\alpha =1-R^{2}$$ (8)

where *Z*_*s*_ (*ω*) is the surface characteristic impedance, *Z*_0_ is the air characteristic impedance and is equal to *ρ*_0_*c*_0_, in which *c*_0_ is the sound speed, and *R* is the sound reflection coefficient.

## 3 Non-acoustical parameter identification model

In the JCA model there are five non-acoustical parameters that need to be identified: porosity, air flow resistivity, tortuosity, viscous characteristic length and thermal characteristic length. In this study, the main task is to identify four parameters except the porosity by means of the optimization techniques as the porosity can be calculated out based on the measured density.

It can be seen from Eq (3) that the viscous characteristic length Λ and thermal characteristic length Λ´ are functions of the shape factor *c* and scale factor *c´* of the pore cross section, respectively. The range of the characteristic length value is commonly from 1 to 3000, whereas the value of shape factor *c* or scale factor *c*´ generally ranges from 0.3 to 3.3. The shape factor *c* and scale factor *c*´ are selected as the design variables instead of the viscous characteristic length and thermal characteristic length because narrowing the solution space helps to converge to a reasonable solution. Set the dimension of the particle *D* to 4, thus the four components in the particle’s position vector represent four unknown parameters in the JCA model, namely ***x*** = [*σ*, *α*_∞_, *c*, *c*´].

It is obvious that the non-acoustical parameter identification is essentially a constrained multi-dimensional parameter optimization problem. The objective is to find the global optimal parameters to make the predicted SAC most consistent with the experimental SAC. According to the principle of the least square method, the fitness function and the constraints can be given in Eq (9).

$$\begin{array}{c} \text{min}\;f_{\text{obj}\;}(f_{i},x)=\sum_{i=1}^{T}{\left[\alpha_{\text{EXP}}(f_{i})-\alpha_{\text{JCA}}(f_{i},x)\right]}^{2}\\ s.t.\{\begin{array}{c} 1000\leq \sigma \leq 200000\\ 1\leq \alpha_{\infty }\leq 4\\ 0.3\leq (c,c^{\prime })\leq 3.3\\ c\geq c^{\prime } \end{array} \end{array}$$ (9)

where *T* is the number of sampling frequency points in the testing frequency range, *f*_*i*_ is the *i*th frequency point sampled in the experiment, *α*_EXP_ denotes the SAC measured at the frequency *f*_*i*_, and *α*_JCA_ denotes the SAC predicted by the JCA model at the same frequency.

## 4 Optimization algorithm

In this section the optimization algorithm adopted to solve the non-acoustical parameter identification model of the porous materials is presented.

### 4.1 Standard particle swarm optimization algorithm

Particle swarm optimization (PSO) algorithm was first proposed by Kennedy and Eberhart in 1995 [19], which is an efficient population-based stochastic search technique. The PSO algorithm regards an individual as a particle without weight or volume in the search space. Each particle in the swarm represents a candidate solution to the optimization problem and flies at a certain speed in the multi-dimensional search space. The flight state can be described by the velocity vector and the position vector. Suppose that in the *D*-dimensional space the current position of *i*th particle (*i* = 1, 2,…, *N*) is ***x***_***i***_ = [*x*_*i*1_, *x*_*i*2_,…, *x*_*iD*_] and the current velocity is ***v***_***i***_ = [*v*_*i*1_, *v*_*i*2_, …, *v*_*iD*_], where *N* denotes the swarm size. The best position encountered by the *i*th particle itself is *p*_*best*_ and the best position in the whole swarm is *g*_*best*_. The position vector of the particle is dynamically adjusted according to its momentum and both the individual and the global memories. The particle therefore takes advantage of the best position to make itself fly towards the optimal solution. Update of the particle velocity and position is written as

$$v_{id}^{k+1}=wv_{id}^{k}+c_{1}r_{1}(p_{id}^{k}-x_{id}^{k})+c_{2}r_{2}(g_{id}^{k}-x_{id}^{k})$$ (10)

$$x_{id}^{k+1}=x_{id}^{k}+v_{id}^{k+1}$$ (11)

where $v_{id}^{k}$ and $x_{id}^{k}$ denote the current velocity and the current position of the *i*th particle at *d*th dimension in the *k*th iteration, $p_{id}^{k}$ and $g_{id}^{k}$ denote *p*_*best*_ and *g*_*best*_ respectively, *w* is the inertia weight which is used to realize the effective control of the particle’s flight velocity, *c*_1_ and *c*_2_ are two acceleration coefficients reflecting the level of self-cognition and social cognition among the particles, *r*_1_ and *r*_2_ are two random numbers uniformly distributed in the interval [0, 1]. Note that, the solution space is bounded by [*x*_min_, *x*_max_] and the velocity is bounded by [*v*_min_, *v*_max_].

### 4.2 Modified particle swarm optimization algorithm

In this subsection, inspired by the previous research work the improvements of the PSO algorithm are made from the following three aspects.

#### 4.2.1 Chaotic initialization

Standard PSO mostly adopts the random distribution strategy to generate the initial population. In the case of a large search space it is difficult for the initial population to give a high ergodic degree, which affects the solving efficiency of the PSO algorithm. To improve the quality of particles, the initial position and velocity are initialized with a pseudo-random chaotic sequence. The chaotic sequences are constructed by the chaotic logistic map, and the map relationship can be expressed as [20]

$$z_{i+1}=\mu \times z_{i}\times (1-z_{i})\;i=0,1,2,\cdots$$ (12)

where *z*_*i*_ denotes the *i*th chaotic variable which is distributed in the interval (0, 1) and *μ* is a predetermined constant called bifurcation coefficient. When *μ*∈[3.57, 4] and *z*_*i*_ ∉{0, 0.25, 0.5, 0.75, 1}, the dynamic system behaves a completely chaotic state [21]. At this time, the track of chaotic variables can be guaranteed to traverse the entire search space. The detailed procedure of the chaotic initialization algorithm in this study is outlined as follows.

***Step 1***. Set iteration number and bifurcation coefficient. Randomly construct a *D* dimensional initial chaotic variable *z*_1_ = [*z*_11_, *z*_12_, …, *z*_1*D*_]; each dimension component is a random number that distributes between 0 and 1.
***Step 2***. If component values of the chaotic variable *z*_*i*_ are 0, 0.25, 0.5, 0.75 and 1, give the component a small perturbation by Eq (13) and then update *z*_*i*_ using Eq (12); otherwise update *z*_*i*_ directly using Eq (12) without any changes.
  $$z_{i}=z_{i}+0.1*r$$ (13)
  where *r* is a random number.
***Step 3***. Suppose *N* is the preset largest iteration times which is equal to the swarm size. If the iteration number *i* = *N*, then stop the iteration; otherwise set *i* = *i*+1, and then go back to step 2.
***Step 4***. After the iteration is completed, the chaotic matrix [*z*_1_; *z*_2_; …; *z*_*N*_] is formed by *N* chaotic vectors. Then remap each element in the matrix from the chaotic region (0, 1) into the initial solution space according to Eq (14).
  $$x_{id}=x_{\text{min}}^{d}+z_{id}(x_{\text{max}}^{d}-x_{\text{min}}^{d}),\;d=1,2,\ldots ,D$$ (14)

#### 4.2.2 Sigmoid-based acceleration coefficients

In order to obtain the balance between the global search competence early in the algorithmic process and the global convergence late in the algorithmic process, literature [22] proposed the adjusting strategy of the acceleration coefficients based on the sigmoid function. The sigmoid-based acceleration coefficients can be written as

$$c_{1}^{k}=\frac{1}{1+\text{exp}(-\lambda \tau )}+2(c_{1f}-c_{1i}){\left(\tau -1\right)}^{2}$$ (15)

$$c_{2}^{k}=\frac{1}{1+\text{exp}(-\lambda \tau )}+(c_{1f}-c_{1i}){\left(\tau \right)}^{2}$$ (16)

where *λ* is the control parameter used to adjust the values of the sigmoid-based acceleration coefficients (*λ* = 0.0001), *c*_1*f*_ and *c*_1*i*_ are constants and the values are 2.5 and 0.5, respectively [22]. *τ* is defined as the ratio of the current iteration times *k* to the maximum iteration times *M*. In the early solution process, when *τ* = 0 the value of *c*_1_ begins to decrease nonlinearly from 2.5 and the value of *c*_2_ begins to increase nonlinearly from 0.5, which makes the initial particles disperse into the solution space. When *τ* = 1, the value of *c*_1_ drops to 0.5 and the value of *c*_2_ rises to 2.5. Under such conditions the tendency for the particles approaching to the optimal position of the group is strengthened.

#### 4.2.3 Adaptive inertia weight

In order to balance the global exploration capacity and the local search optimum capacity, the adaptive inertial weight factor strategy is adopted to dynamically change the inertia weight according to the fitness values. The adaptive inertial weight factor is often used in conjunction with the chaotic sequence to improve the performance of searching optimum [23]. The inertia weight can be expressed as

$$w_{i}^{k}=\{\begin{array}{ll} w_{\text{min}}+\frac{\left(w_{\text{max}}-w_{\text{min}})(f_{i}^{k}-f_{\text{min}}^{k}\right)}{f_{\text{avg}\;}^{k}-f_{\text{min}}^{k}} & ,f_{i}^{k}\leq f_{\text{avg}\;}^{k}\\ w_{\text{max}} & ,f_{i}^{k}>f_{\text{avg}\;}^{k} \end{array}$$ (17)

where $w_{i}^{k}$ and $f_{i}^{k}$.represent the inertial weight and fitness value of the *i*th particle in the *k*th iteration, *w*_max_ and *w*_min_ are the maximal and the minimal values of the inertial weight, $f_{\text{avg}}^{k}$ and $f_{\text{min}}^{k}$ denote the average fitness value and the minimum fitness value of current total particles, respectively. If the fitness value is smaller than its average value, a relatively small *w* is given to slow down the velocity of the particles in local space to find the global optimal solution. If the fitness value is larger than its average value, the step length of searching optimum needs to be increased to improve the capacity of global searching optimum by setting the inertial weight value as the maximum *w*_max_.

### 4.3 Implementation of the modified particle swarm optimization algorithm

**Fig 1:**
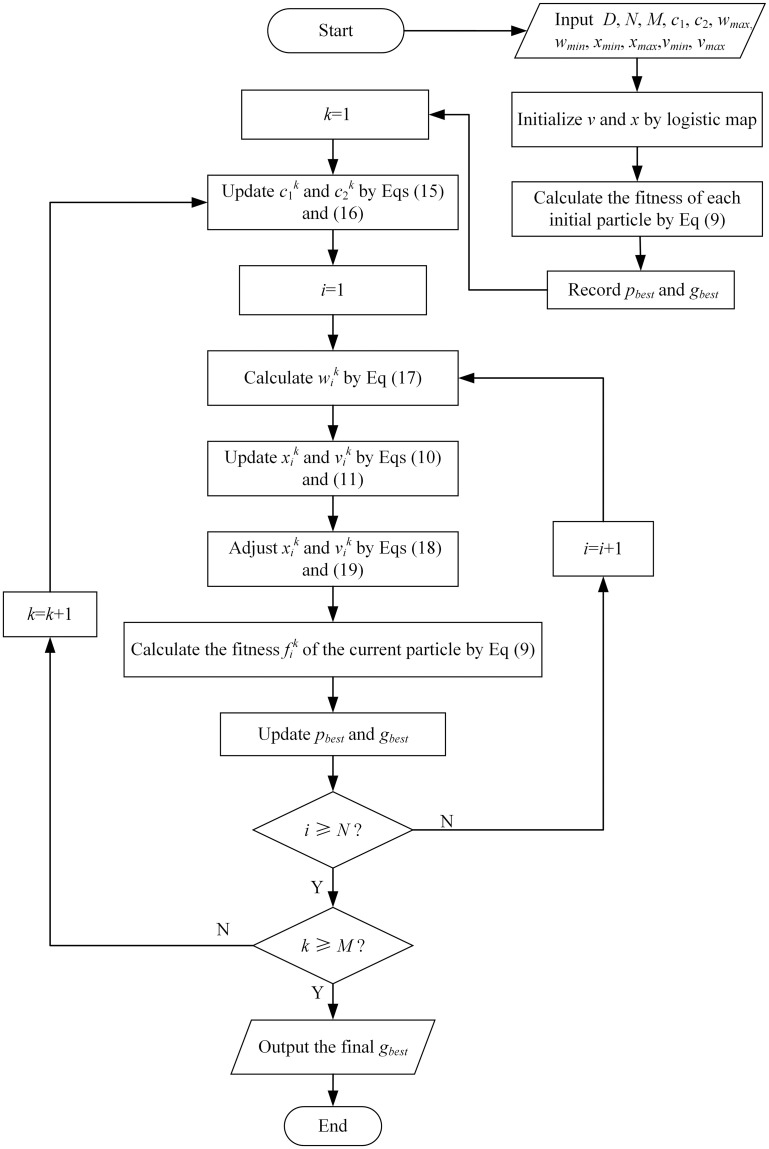
Flow chart of the MPSO algorithm.

The flow chart of the MPSO algorithm is shown in Fig 1 and the detailed steps of the algorithm are summarized as follows.

***Step 1***. Input parameter values to the algorithm, including *D*, *N*, *M*, *w*_max_, *w*_min_, *x*_min_, *x*_max_, *v*_min_ and *v*_max_.
***Step 2***. Initialize velocity and position of the particles based on the chaotic initialization algorithm mentioned above, then calculate the fitness for all particles in current population according to Eq (9). At the same time record the current optimal position of the individual particle *p*_*best*_ and the global optimal position *g*_*best*_.
***Step 3***. Set *k* = 1.
***Step 4***. Determine $c_{1}^{k}$ and $c_{2}^{k}$ by Eqs (15) and (16).
***Step 5***. Set *i* = 1.
***Step 6***. Calculate $f_{avg}^{k}$ and $f_{\text{min}}^{k}$ of current particle population.
***Step 7***. Determine $\omega_{i}^{k}$ by Eq (17).
***Step 8***. Update the velocity $v_{i}^{k}$ and position $x_{i}^{k}$ of the particles based on Eqs (10) and (11).
***Step 9***. If the position and velocity are out of the range, adjust them by using Eqs (18) and (19).
  $$x_{id}^{k}=\{\begin{array}{cc} x_{\text{min}}^{d}, & x_{id}^{k}<x_{\text{min}}^{d}\\ x_{id}^{k}, & x_{\text{min}}^{d}\leq x_{id}^{k}\leq x_{\text{max}}^{d}\\ x_{\text{max}}^{d}, & x_{id}^{k}>x_{\text{max}}^{d} \end{array}$$ (18)
  $$v_{id}^{k}=\{\begin{array}{cc} v_{\text{min}}^{d}, & v_{id}^{k}<v_{\text{min}}^{d}\\ v_{id}^{k}, & v_{\text{min}}^{d}\leq v_{id}^{k}\leq v_{\text{max}}^{d}\\ v_{\text{max}}^{d}, & v_{id}^{k}>v_{\text{max}}^{d} \end{array}$$ (19)
***Step 10***. Calculate the fitness $f_{i}^{k}$ of the current particle by Eq (9).
***Step 11***. Update *p*_*best*_ and *g*_*best*_.
***Step 12***. If *i* < *N*, set *i* = *i*+1 and go back to step 7, otherwise go to the next step.
***Step 13***. If *k* < *M*, set *k* = *k*+1 and go back to step 4, otherwise terminate the algorithm and output the final optimal solution *g*_*best*_ according to the minimum fitness value.

Run the MPSO algorithm many times and then determine the final optimal results according to the smallest fitness value. The optimal viscous characteristic length and thermal characteristic length can be calculated through the shape factor *c* and the scale factor *c´* according to Eq (3). Thus the JCA prediction model with identified parameters is determined.

### 4.4 Verification of the modified particle swarm optimization algorithm

Verification of the feasibility for the MPSO algorithm applied to parameter identification of porous materials is carried out based on the relevant data offered by literature [24]. In literature [24], analytical method, indirect method, genetic algorithm and iterative method were all used to predict the five non-acoustical parameters of a polyurethane foam, respectively. The measured values of the air flow resistivity, porosity and tortuosity of this material were listed in Table 1. Literature [25] used the method of multi-levels inverse estimation to obtain the five non-acoustical parameter values for the same material. In this study the MPSO algorithm is also utilized to estimate the five parameters of the same material. Set the maximum iteration number *M* as 150 and the population size *N* as 50. The *w*_min_ and *w*_max_ of the inertial weight are set as 0.4 and 0.9, respectively. The parameter values identified by the above mentioned methods are listed in Table 1.

**Table 1:** Parameter values measured and identified by the above mentioned methods.

Parameters	Air flow resistivity *σ* (N·s/m^4^)	Porosity *ϕ*	Tortuosity *α*_*∞*_	Viscous characteristic length Λ (μm)	Thermal characteristic length Λ’ (μm)
Methods					
Measured-[24]	5359	1	1.08	-	-
Analytical method-[24]	6641	0.9	1.03	83	267
Indirect method-[24]	6414	0.99	1.15	135	250
Genetic algorithm-[24]	6252	0.99	1.14	132	251
Iterative method-[24]	6200	0.99	1.10	130	250
Multi-levels inverse estimation-[25]	5658	0.98	1	100	250
MPSO algorithm	5340	0.99	1.12	121	271

In Table 1, the relative errors of the air flow resistivity identified by the methods addressed in literature [24] are all more than 15%. The relative error of air flow resistivity identified by the method of multi-levels inverse estimation is reduced to 5.2%, while the relative errors of the porosity and the tortuosity are increased obviously [25]. However, for the MPSO algorithm, the relative error of the air flow resistivity is reduced to 0.35%, and the relative errors of the porosity and the tortuosity are 1% and 3.70%, respectively. It is obvious that among the above methods the MPSO algorithm has the most advantages in identifying the non-acoustical parameters involved in the JCA model of the porous materials.

## 5 Application of modified particle swarm optimization algorithm

In this section the MPSO algorithm is adopted to identify the non-acoustical parameters involved in the JCA model of the jute fiber felt based on the SAC measured by the impedance tube. The SAC predicted by the JCA model is compared with the experimental SAC. And the identification performance and computation performance of the MPSO algorithm are discussed.

### 5.1 Jute fiber felt sample

**Fig 2:**
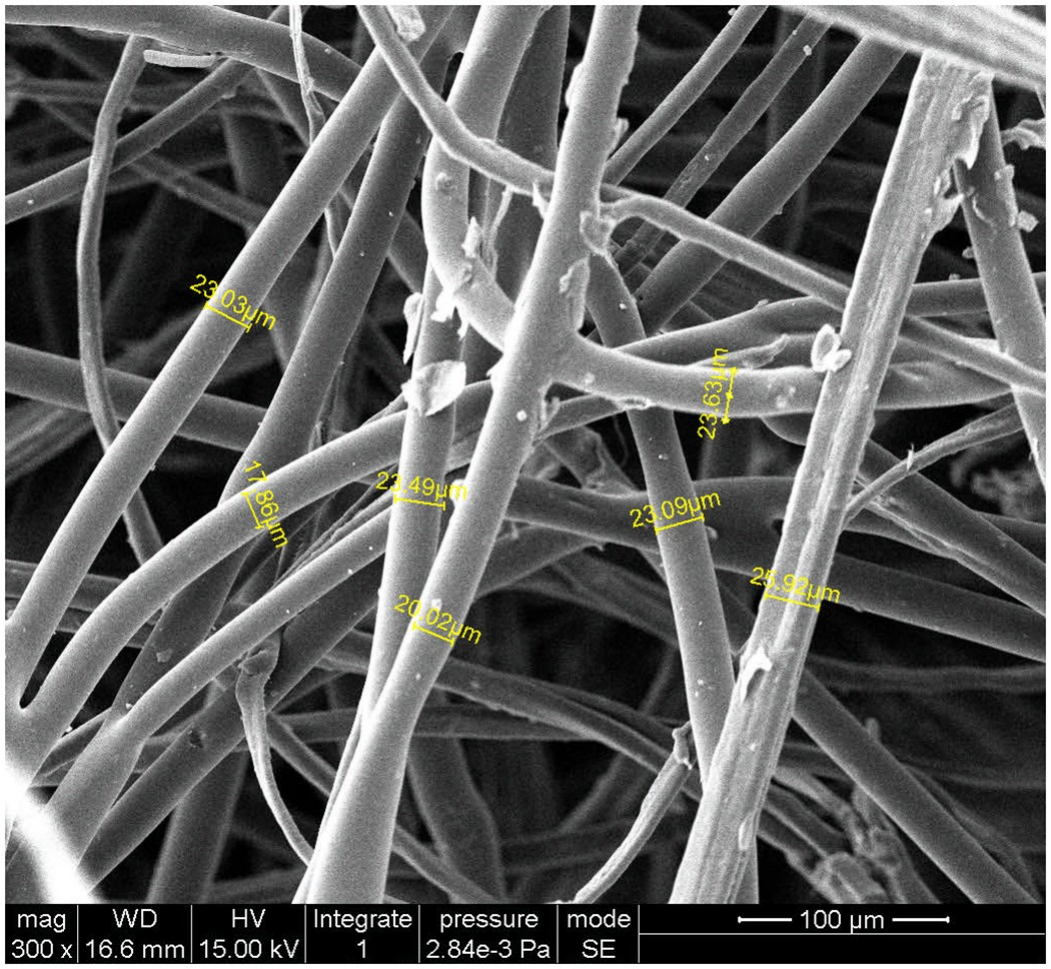
SEM photograph of the jute fiber felt.

Natural fibers have better sound absorption performance due to their naturally formed porous structure [8]. Jute fiber is a kind of natural fiber with excellent performance and it is commonly used as vehicle ceiling, door interior frame, seat back, and other interior trim substrates and acoustic packaging materials [26]. The jute fiber is stacked, heated and bonded into a felt-like form after the mixing, carding and net-paving process. The microscopic structure under environmental scanning electron microscopy of the jute fiber felt is shown in Fig 2, and the statistical average of the fiber diameter is 23.67 μm.

The porosity of the jute fiber felt can be estimated according to Eq (20).

$$\phi =(1-\rho_{m}/\rho_{f})\times 100\text{\%}$$ (20)

in which, *ρ*_*m*_ is the density of the jute fiber felt sample, *ρ*_*f*_ is the density of the raw material and the porosity of the jute fiber felt comes out to be 0.96.

The jute fiber felt is made into two circular samples with different diameters. Their diameters match with the inner diameters of the large and small impedance tubes, respectively. The geometric and physical parameters of the two samples are listed in Table 2, where *L* and *S* indicate the large-diameter and the small-diameter samples, respectively.

**Table 2:** Geometric and physical parameters of the samples.

Parameters	Sample *L*	Sample *S*	Average
**Diameter (mm)**	100.92	31.5	-
**Thickness (mm)**	18.93	18.65	18.79
**Density (kg/m**^**3**^**)**	43.14	40.72	41.93
**Porosity**	0.96	0.96	0.96

### 5.2 Testing of the sound absorption coefficient for the jute fiber felt

**Fig 3:**
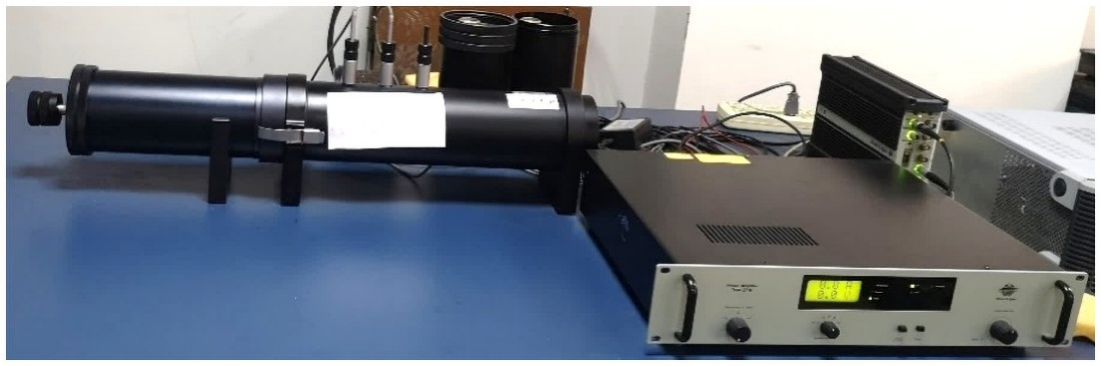
SAC testing system of the jute fiber felt.

**Fig 4:**
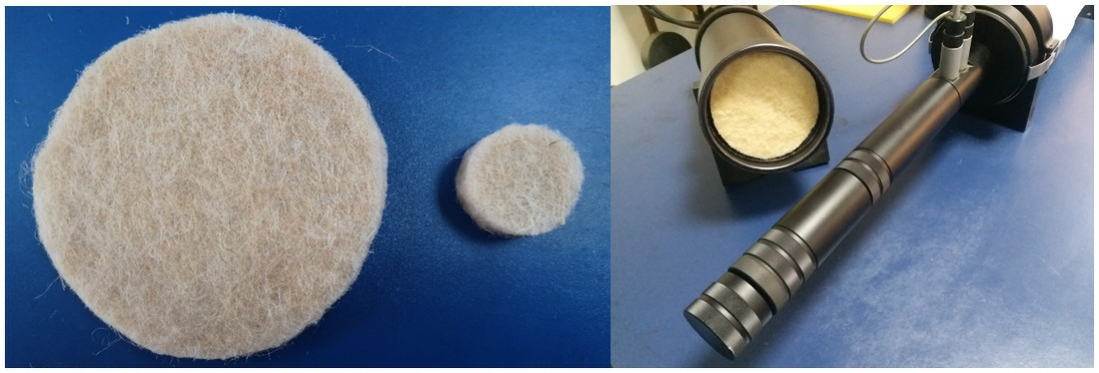
Jute fiber felt samples and the impedance tube installed with a sample.

In order to obtain the SAC curve of the jute fiber felt the SAC testing system shown in Fig 3 is established, which consists of two sets of B&K 4206 impedance tubes, the power amplifier, the sound calibration instrument and the PULSE analysis software, etc. Two jute fiber felt samples and the impedance tube installed with a sample are displayed in Fig 4.

The acoustic impedance tube test system is used to measure the SAC of the samples according to the ISO 10534–2: 1998 [27]. The large diameter impedance tube is used to measure the SAC of the sample at the frequency ranging from 250 Hz to 1600 Hz, and the small diameter impedance tube is applied to the frequency ranging from 500 Hz to 6000 Hz. It is obvious that there is an overlap of the SAC values at the frequency ranging from 500 Hz to 1600 Hz. The SAC values in the overlap frequency range can be calculated according to Eq (21) [28].

$$\alpha_{\text{total}}=(1-\frac{f-500}{1100})\alpha_{S}+\frac{f-500}{1100}\alpha_{L}$$ (21)

where *α*_*S*_ and *α*_*L*_ represent the SAC measured by the small tube and the large tube, respectively.

### 5.3 Results and discussion

In this subsection the identification performance and the computational performance of the MPSO algorithm are discussed.

#### 5.3.1 Comparison of the identification performance

Based on the experimental SAC and the porosity of the jute fiber felt, the standard PSO algorithm and the MPSO algorithm are adopted to identify the non-acoustical parameters involved in the JCA model of the jute fiber felt. Set the maximum iterative number *M* as 150 and the population size *N* as 50. The *w*_min_ and *w*_max_ of the inertial weight are set as 0.4 and 0.9, respectively. In the standard PSO algorithm, *c*_1_ and *c*_2_ are both set as 2 and the inertial weight reduces linearly [29]. The rest parameters of the PSO algorithm are set the same as those of the MPSO algorithm. The optimization process runs ten times independently. The optimal parameter values and the fitness values are listed in Table 3.

**Table 3:** Comparison of the identification results by the PSO and MPSO algorithms.

Algorithms	Fitness values				Optimal non-acoustical parameter values (Min.)			
	Min.	Max.	Avg.	Std.	Air flow resistivity *σ* (N·s/m^4^)	Tortuosity *α*_∞_	Viscous characteristic length Λ (μm)	Thermal characteristic length Λ´ (μm)
PSO	0.8411	89.1185	46.6094	38.6162	13629	1	353	353
MPSO	0.2497	2.4874	1.1448	1.1555	12742	1	267	267

It can be seen from Table 3 that the average fitness value of MPSO algorithm is much lower than that of the PSO algorithm. For the MPSO algorithm the difference between the maximum and the minimum fitness values is not significant, which demonstrates the optimization process is stable. The excellent performance of the MPSO algorithm is mainly attributed to the improvements of the PSO algorithm in three aspects. The chaotic initialization mechanism enables the MPSO algorithm to generate a diverse initial population before entering iteration. Both the sigmoid-based acceleration coefficient and the adaptive inertia weight factor are conducive to the emergence of the optimal solution and the stability of the MPSO algorithm.

**Fig 5:**
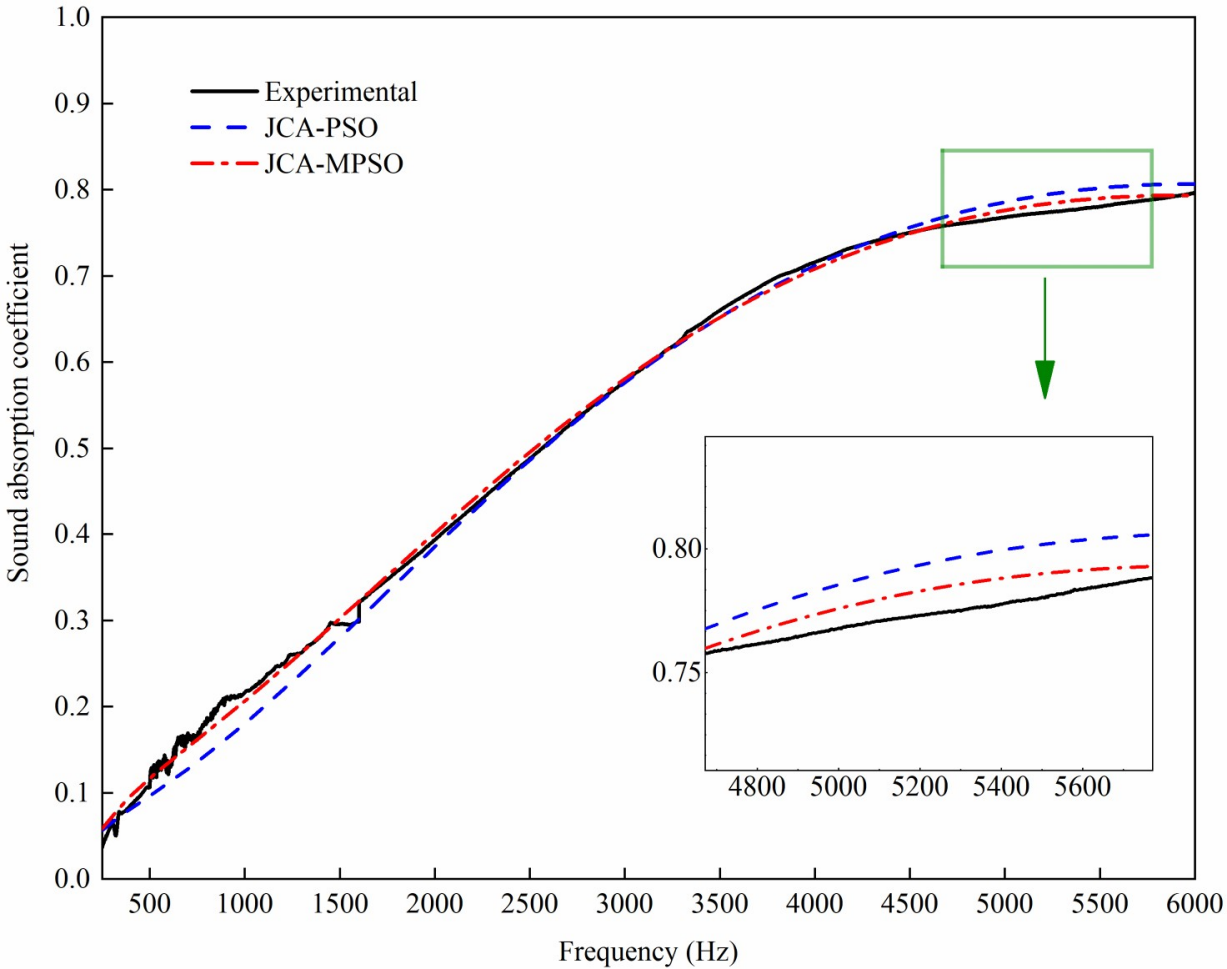
Comparison of the three SAC curves of the jute fiber felt.

Substitute the values of the non-acoustical parameters shown in Table 3 into the JCA model to predict the SAC of the jute fiber felt. The predicted SAC curve and the experimental curve are shown in Fig 5. As can be observed from Fig 5, the jute fiber felt has excellent sound absorbing performance in a wide frequency range and the peak value of SAC can reach 0.8. Compared with the SAC curve predicted by the PSO algorithm, the SAC curve predicted by the MPSO algorithm has better consistency with the experimental curve. Therefore, it demonstrates that the MPSO algorithm has more advantages in predicting the non-acoustical parameters of the JCA model for the porous materials.

#### 5.3.2 Comparison of the computational performance

**Fig 6:**
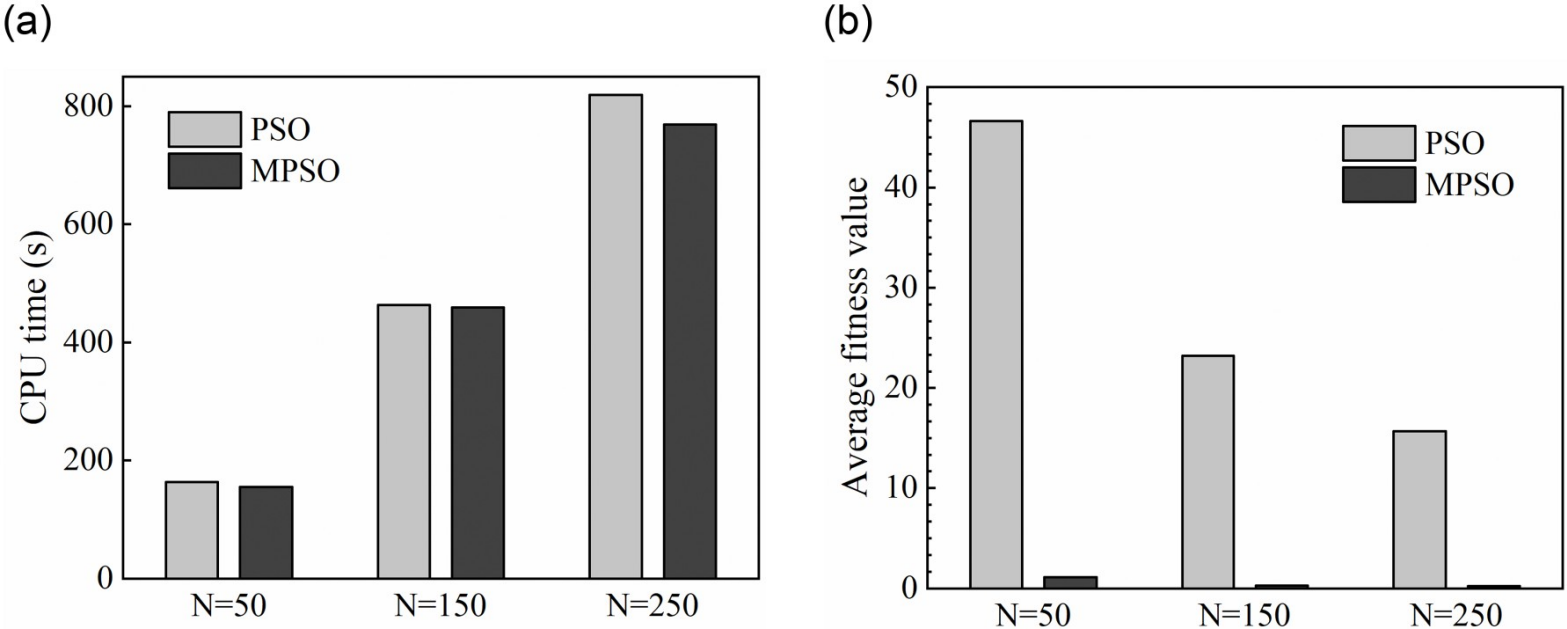
CPU time and average fitness value for two algorithms at the case of *N* = 50, 150 and 250. **(*a*)** CPU time. **(*b*)** Average fitness value.

**Fig 7:**
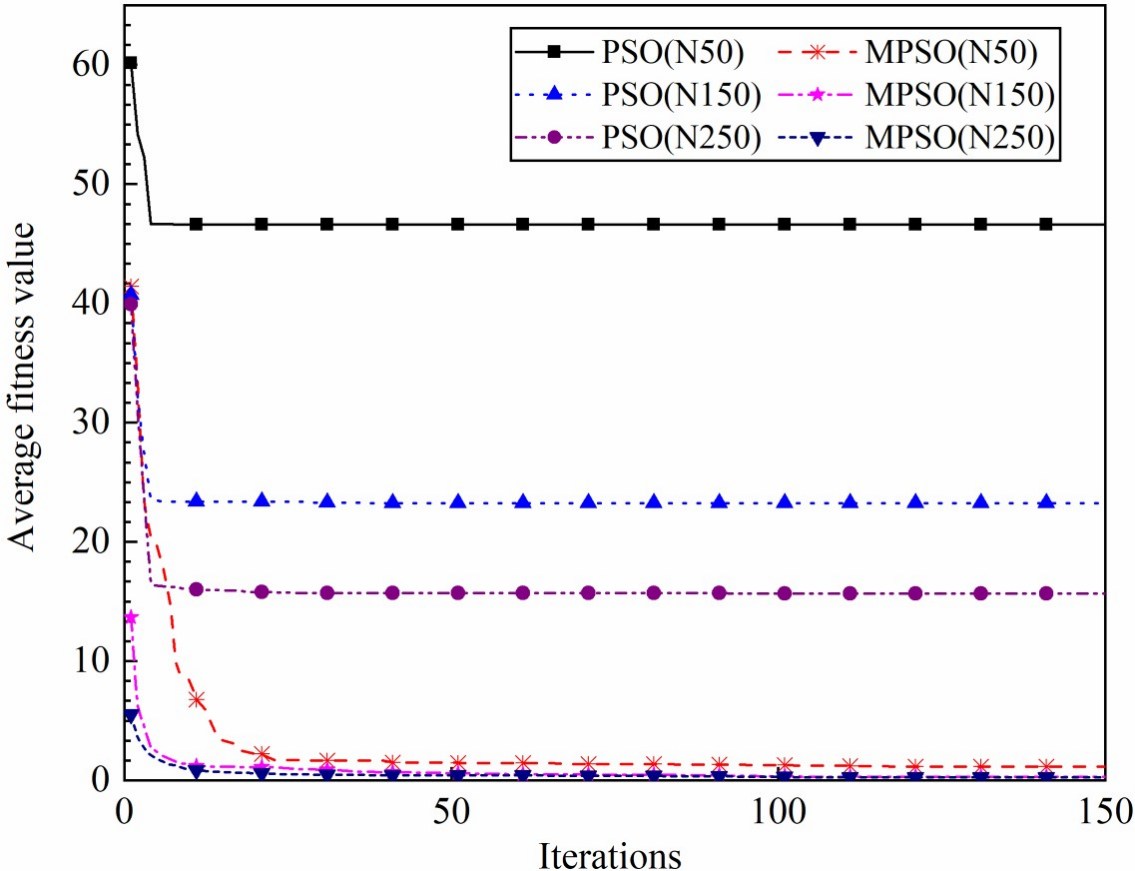
Iterative results of PSO and MPSO algorithms.

In order to verify the efficiency of the MPSO algorithm and analyze the influence of population size on the algorithm performance, two cases are tested with different population size (*N* = 50, 100, 150). Results of the comparison are shown in Figs 6 and 7.

It can be seen from Fig 6(a) that the CPU time costed by the two algorithms increases with the population size, which is caused by the increase of computational effort. With the same population size the MPSO algorithm takes the shorter CPU running time which is 4.2% average less than the running time taken by the PSO algorithm. With the increase of population size, the MPSO algorithm has more advantages in terms of running speed. As shown in Fig 6(b), the optimal average fitness values of the MPSO and PSO algorithms decrease with the increasing of the population size. Moreover, for three different population size the average fitness values of the MPSO algorithm are very small, which indicates that the MPSO algorithm has more advantages in searching the global optimal solution.

As can be seen from Fig 7, the average fitness value of the PSO algorithm decreases quickly at the beginning of iteration and the activity of the population particles reduces after a few times of iteration, which leads the algorithm to converge quickly and the optimal solution tends to fall into the local optimum. And the smaller the population size is, the more likely it is to fall into local optimum. It means that for the case of standard PSO algorithm in order to obtain better identification performance for the non-acoustical parameters it needs larger population size, while larger population size means higher computing time cost. Compared with the PSO algorithm the average fitness value of the MPSO algorithm decreases slowly at the beginning of iteration and the curves maintain downward trend even in the later period of iteration, which demonstrates the strong global search capability and avoids the problem of premature convergence. Moreover, the average fitness value of MPSO algorithm is not easily affected by the population size. Even if the population size is smaller the MPSO algorithm can still obtain the better solution. In other words, the MPSO algorithm can achieve the global minimum with high tolerance for the variations of the population size and the control parameters. The MPSO algorithm presents good performance in the identification of the non-acoustical parameters for the natural porous materials.

## 6 Conclusions

In this study, a MPSO algorithm is adopted to identify the non-acoustical parameters involved in the sound absorption model of the porous materials. The feasibility of the MPSO algorithm applied to the non-acoustical parameter identification of porous materials is investigated. The identification performance and the computational performance of the MPSO algorithm in identifying the non-acoustical parameters of the jute fiber felt are discussed. Research results show that the MPSO algorithm can accurately and effectively identify the non-acoustical parameters involved in the JCA model of the porous materials. Compared with the standard PSO algorithm the SAC curve predicted by the MPSO algorithm has better consistency with the experimental SAC curve, and in terms of the computer running time the MPSO algorithm costs shorter time, especially when the population size increases the MPSO algorithm presents more obvious advantages. In addition, this study demonstrates that the jute fiber felt is a good acoustical green fibrous material which has excellent sound absorbing performance in a wide frequency range and the peak value of its SAC can reach 0.8.

This study is limited in a specific condition, i.e., variability is not considered properly. Future research could analyze the effect of different algorithm parameters on the solution quality and convergence speed, which is conducive to demonstrating the algorithm robustness and obtaining more effective parameter design. Future possible work is to propose more efficient evolutionary algorithms to solve parameter identification problems with more practical constraints.

## Supporting information

S1 Data (XLSX)

S2 Data (XLSX)
